# Risk for SARS-CoV-2 infection in healthcare workers outside hospitals: A real-life immuno-virological study during the first wave of the COVID-19 epidemic

**DOI:** 10.1371/journal.pone.0257854

**Published:** 2021-09-28

**Authors:** Maria Fröberg, Sadaf Sakina Hassan, Ville N. Pimenoff, Susanne Akterin, Kalle Conneryd Lundgren, K. Miriam Elfström, Joakim Dillner

**Affiliations:** 1 Gustavsberg Primary Care Center, Region Stockholm, Gustavsberg, Sweden; 2 Akademiskt Primärvårdscentrum, Region Stockholm, Stockholm, Sweden; 3 Department of Laboratory Medicine, Karolinska Institute, Stockholm, Sweden; 4 Karolinska University Hospital, Stockholm, Sweden; 5 Medical Diagnostics Karolinska, Karolinska University Hospital, Stockholm, Sweden; University of Helsinki: Helsingin Yliopisto, FINLAND

## Abstract

**Objectives:**

Most COVID-19 related infections and deaths may occur in healthcare outside hospitals. Here we explored SARS-CoV-2 infections among healthcare workers (HCWs) in this setting.

**Design:**

All healthcare providers in Stockholm, Sweden were asked to recruit HCWs at work for a study of past or present SARS-CoV-2 infections among HCWs. Study participants This study reports the results from 839 HCWs, mostly employees of primary care centers, sampled in June 2020.

**Results:**

SARS-CoV-2 seropositivity was found among 12% (100/839) of HCWs, ranging from 0% to 29% between care units. Seropositivity decreased by age and was highest among HCWs <40 years of age. Within this age group there was 19% (23/120) seropositivity among women and 11% (15/138) among men (p<0.02). Current infection, as measured using PCR, was found in only 1% and the typical testing pattern of pre-symptomatic potential “superspreaders” found in only 2/839 subjects.

**Conclusions:**

Previous SARS-CoV-2 infections were common among younger HCWs in this setting. Pre-symptomatic infection was uncommon, in line with the strong variability in SARS-CoV-2 exposure between units. Prioritizing infection prevention and control including sufficient and adequate personal protective equipment, and vaccination for all HCWs are important to prevent nosocomial infections and infections as occupational injuries during an ongoing pandemic.

## Introduction

The first wave of the COVID-19 epidemic started in March 2020 in Sweden, [1] with the Stockholm region particularly impacted. There has so far 1,123,413 individuals with SARS-CoV-2 infection and 14 685 COVID-19 deaths in Sweden [2]. In February 2020, the WHO published guidelines on occupational safety for healthcare workers since they are in the front line of any outbreak [3]. The guidelines names employers and managers in health facilities as responsible for the occupational safety of employees including providing adequate training, infection prevention and control (IPC) and personal protective equipment (PPE).

Yet, in May 2020, about half of all confirmed cases of COVID-19 in Sweden were among healthcare workers (HCWs) [4]. Compared to many other countries [5, 6] recommendations for IPC were rather limited in Sweden. HCWs were not systematically tested for SARS-CoV-2 and wearing of face masks not generally recommended neither for HCWs nor for patients [7]. People in need of nursing and/or medical care on a regular basis were therefore to a large extent unprotected. The setting thus approximates a natural history study of the infection. By the end of May 2020, in the Stockholm region (with a population of about 2,300,000 inhabitants) about 2,127 individuals with COVID-19 had died and 806 had been admitted to the intensive care unit (ICU) [8]. During the first wave there were 4,499 deaths due to COVID-19 in the whole Sweden [4]. Of these, 2,257 (50%) deaths occurred at care homes [9].

Rapid community transmission of COVID-19 was typical for the local outbreaks in Sweden and asymptomatic healthcare workers (HCW) in primary care, home healthcare, and palliative care likely had a distinctive role in the transmission events [3, 6, 10–12]. Local rapid community transmission was most evident in local care homes, where a majority of COVID-19 deaths occurred in Stockholm during the first outbreak [4, 9]. Although the Swedish Public Health Agency recommended that all healthcare staff with symptoms suspicious of COVID-19 should be tested for SARS-CoV-2, testing was low or absent during the first wave [13]. During the first wave of the epidemic in Sweden, testing for presence of SARS-CoV-2 was limited to patients admitted to hospitals. Ambulatory testing for suspected cases of COVID-19 at care homes was accessible and requested by physician in charge. To some extent, ambulatory testing was available for patients with co-morbidities in home-based care. PPE was generally scarce in Swedish healthcare and care homes received insufficient PPE for their staff [9, 14]. Simultaneously, primary care centers made medical assessments of patients with suspected COVID-19 at their outpatient infection clinics using IPC precautions, but without having access to testing. Healthcare environment related infections not only endanger the patient but risk the healthcare personnel, and further, can quickly limit the healthcare units care capacity. Accumulating evidence indicate that staff in primary care, at homes and in palliative care were highly exposed to SARS-CoV-2 infections, and that a large fraction of the infections were asymptomatic [10–12, 15, 16]. Therefore, it is important to efficiently detect and manage healthcare-related SARS-CoV-2 exposures. We have previously found that presymptomatic infection can be readily detected by SARS-CoV-2 antibody screening and SARS-CoV-2 RT-PCR testing [10, 11]. Seropositive HCW have no excess risk for disease in the coming months [11]. Subjects who test positive for high amounts (low Cycle Threshold value) of the SARS-CoV-2 viral genome in nasopharyngeal swabs have a very high likelihood to become sick in the near future [10]. The epidemic is known to be in particular driven by so-called “superspreaders” who are commonly not yet symptomatic (pre-symptomatic) [10]. To assess how frequent pre-symptomatic disease was in comparison to the cumulative exposure to SARS-CoV-2 infections among HCWs in primary care, at homes and in palliative care in Stockholm during the March-June 2020 SARS-CoV-2 outbreak, we recruited most of the healthcare personnel for SARS-CoV-2 antibody screening and SARS-CoV-2 RT-PCR testing and compared the results to a large cohort of hospital HCWs that was tested in exactly the same manner.

## Material and methods

### Study design and subjects

In May 2020, we asked the healthcare providers in Stockholm, Sweden to invite their employees to a study of past or present SARS-CoV-2 infections in the healthcare setting. The HCWs enrolled were from all professions within healthcare. The Stockholm Region is a relatively densely populated region in Sweden: the region has 2,34 million inhabitants, out of Sweden’s 10.23 million inhabitants (according to public data from 2019). The units who participated were 22 primary care centers, 2 care homes, and one palliative care unit representing approximately 1000 employees. Recruitment was carried out in June 2020. Healthcare staff on duty (hence without any disease symptoms) were eligible. To be included in the study, each study subject needed to provide a written informed consent form. For each unit included in the study, there also needed to be an approval by the operations manager. Staff members were excluded if they were absent from work or had an incomplete consent form. Among the included 839 individuals, swab samples for PCR were missing for 60 and invalid for 39 individuals. If one of the samples was present, the study subject was included in the study. If both samples were invalid or absent, the individual was excluded. Operations managers also reported measures taken to limit transmission of SARS-CoV-2 (S1 File).

### Methods

Viral RNA detection and antibody detection methods were as described [10]. Briefly, viral RNA was extracted from throat swab samples using MGIEasy Magnetic Beads Virus DNA/RNA extraction kit and subjected to real-time PCR. Serological analysis (99.2% sensitivity and 99.8% specificity assay) was performed by IgG detection in inactivated serum. Antibodies were tested against three different variants of viral proteins (Spike trimers containing prefusion-stabilized spike glycoprotein ectodomain, Nucleocapsid protein and Spike S1 domain) and reactivity against at least two viral antigens was required for a sample to be assigned as IgG positive. SARS-CoV-2 testing results (PCR and serology) were reported as proportions by workplace, age, and sex. A one-sided Cochran Armitage test for trend was used to examine patterns in serology prevalence by age. The analyses were done in SAS 9.4, Cary NC, USA.

### Ethics declaration

The approval of the study was granted by the National Ethical Review Agency of Sweden (Decision number 2020–01620 and 2020–01881). Trial registration number: ClinicalTrials.gov NCT04411576. All methods in the study were carried out in compliance with the Helsinki declaration.

## Results

A total of 25 different care units joined our study, from which 839 employees were enrolled (83% out of 1009 employees in total). The size of the workplaces varied as well as the participation rate at each workplace. The median age of employees was 47. Overall, 8/839 (1%) were PCR positive and 100/839 (12%) of employees were serology positive at the time of sampling. Only three individuals were serology negative and PCR positive. Only two of these three individuals had the typical potential “presymptomatic test result” with large amounts of viral nucleic acids in their nasopharyngeal swabs (CT value below 27.0 in the PCR). PCR positivity ranged from 0–7% among the different workplaces and serology positivity ranged from 0–29% (6 workplaces had a serology prevalence among employees of over 20%) (Table 1). Test positivity, both PCR and serology, did not differ overall by sex but there tended to be differences in certain age-strata. The prevalence of serology positivity was higher for women ages 20–40 than men. Within this age group there was 19% (23/120) seropositivity among women and 11% (15/138) among men (p<0.02). In the ages 50 and above while it was higher for men as compared to women. There was a trend of decreasing serology positivity by age (one sided p-trend <0.05) (Table 2). We performed a large parallel study of almost 10,000 hospital HCWs employed at the Karolinska University Hospital, that has been published separately [10].

**Table 1:** Descriptive statistics for workplaces included in the study and distribution of SARS-CoV-2 testing results by workplace.

Workplace	Employees (total) n	Participating employees n	Participation rate (%)	Age (median, min-max) years	PCR positive n (%)	Serology positive n (%)
**Primary Health Care (PHC)**						
Alby PHC	32	27	84%	47; 23–71	2 (7%)	5 (19%)
Axelsbergs PHC	43	25	58%	39; 28–67	0	2 (8%)
Bredäng PHC	22	24	109%*	45.5; 24–66	0	1 (4%)
Danviken	14	14	100%	46; 28–73	0	4 (29%)
Djurö PHC	21	20	95%	50; 27–67	1 (5%)	1 (5%)
Ektorps PHC	40	31	78%	49; 25–64	0	3 (10%)
Familjeläkarna	287	128	45%	41; 19–68	1 (1%)	20 (16%)
Fisksätra PHC	17	12	71%	56,5; 39–68	0	1 (8%)
Fittja PHC	26	22	85%	49; 28–70	0	2 (9%)
Flemingsbergs PHC	42	29	69%	41; 24–63	0	4 (14%)
Gustavsbergs PHC	150	150	100%	49; 24–76	1 (1%)	13 (9%)
Helsa PHC, Älta	26	27	104%*	41; 21–69	0	2 (7%)
Kvarnholmens PHC	9	9	100%	45; 38–67	0	2 (22%)
Salems PHC	Unknown	47	Unknown	49; 22–65	0	5 (11%)
Sickla Hälsocenter	40	32	80%	48.5; 30–68	1 (3%)	1 (3%)
Storvretens PHC	30	7	23%	52; 29–62	0	1 (14%)
Sätra PHC	18	17	94%	42; 23–67	0	0
Tullinge PHC	50	41	82%	49; 30–64	0	10 (24%)
Tumba PHC	40	43	108%*	50; 27–68	0	5 (12%)
Tungelsta PHC	14	8	57%	43,5; 35–63	0	2 (25%)
Vaxholms PHC	25	14	56%	48; 20–61	0	3 (21%)
Värmdö PHC	17	17	100%	50; 31–71	0	1 (2%)
**Care homes**						
Attendo Lindhovshemmet	Unknown	41	Unknown	48; 23–70	1 (2%)	2 (5%)
Attendo Solbacken	Unknown	30	Unknown	45,5; 25–65	0	1 (3%)
**Palliative care unit**						
Maria Regina Hospice	50	24	48%	51,5; 32–71	1 (4%)	7 (29%)
**Total**	1009	839	83%	47; 19–76	8 (1%)	98 (12%)

*Participation rate over 100% can be due to inclusion of extra staff and/or variations in the exact number of employees over time.

**Table 2:** Distribution of SARS-CoV-2 testing results by age and sex.

Age*	Participants n	PCR positive n (%)	Serology positive n (%)
**All ages**	**839**	**8 (1%)**	**100 (12%)**
Women	434	2 (0%)	51 (12%)
Men	405	6 (1%)	49 (12%)
**<30 years**	**59**	**0 (0%)**	**9 (15%)**
Women	32	0 (0%)	7 (22%)
Men	28	0 (0%)	2 (7%)
**30–39 years**	**199**	**4 (2%)**	**29 (15%)**
Women	88	1 (1%)	16 (18%)
Men	111	3 (3%)	13 (12%)
**40–49 years**	**227**	**3 (1%)**	**24 (11%)**
Women	119	1 (1%)	12 (10%)
Men	108	2 (2%)	12 (11%)
**50–59 years**	**229**	**1 (0.4%)**	**28 (12%)**
Women	138	0 (0%)	14 (10%)
Men	91	1 (1%)	14 (15%)
**60+ years**	**125**	**0 (0%)**	**10 (8%)**
Women	57	0 (0%)	2 (4%)
Men	68	0 (0%)	8 (12%)

*One-sided Cochran Armitage test for trend by age and serology prevalence, p-value = 0.0467.

## Discussion

### Main findings

Our most important finding is that young HCWs in primary care, at homes and in palliative care, particularly young women, had frequently had SARS-CoV-2 infection, as demonstrated by the over 22% seroprevalence among female HCWs in ages 20–29. This is a significant finding because it suggests that young HCWs who are at the front line in primary care and other care settings outside hospitals may have been a source of nosocomial infections. We found a low prevalence of PCR positivity (1%) mostly coupled with serology positivity, suggesting that the PCR positivity was mostly post-symptomatic (the affected HCWs may have returned to work after symptoms have resolved). The finding of many more HCWs with past infection than with pre-symptomatic infection indicates that the study was performed at a time when the first wave of the epidemic was already subsiding in the region. Healthcare workers in primary care, in care homes and in palliative care are particularly responsible for and in close and frequent contact with older people and chronically ill patients with complex care needs who are vulnerable to COVID-19. During the first wave of the COVID-19 pandemic, in March to June 2020, in Sweden, protective measures for healthcare workers in Sweden were limited: postponing meetings that could wait, keeping a safe distance when possible, limit the time of close contact with care takers and to wear a surgical mask, a gown and gloves—but only if the patient had symptoms suggestive of COVID-19. Testing and contact tracing of patients with possible COVID-19 was not available for these settings during the first wave. These circumstances made it quite difficult for healthcare workers to effectively protect older and vulnerable people from COVID-19 –and to protect themselves. The data in the present study thus probably reflect a situation that would be close to a natural history setting.

The typical testing results found among subjects who will have symptomatic disease in the future (pre-symptomatic subjects; PCR positivity for high amounts of virus (CT value <27) and lack of antibodies) was found in only 2/839 subjects, considerably lower than in a major hospital in the same region tested a few weeks earlier (57/9449 subjects) [10] also implying that the number of new infections was declining at the time of the present study. The seroprevalence of SARS-CoV-2 specific antibodies varied substantially between units (0–29%). Hence, it is possible that cluster outbreaks may have occurred within units and is in line with previous literature on the spread of SARS-CoV-2 among HCWs [6, 12, 17, 18]. This finding is also in line with our result that the typical pattern of potential presymptomatic (“Superspreading”) HCWs was rare in our cohort.

Strengths of the study include the fact that it is a rather large study with a high attendance rate among HCWs in areas of healthcare that were of particular importance in the epidemic. Furthermore, PCR testing and serology testing was performed simultaneously with advanced technology. Weaknesses of the study include the fact that it was launched at a time when most of the first wave of the epidemic was already over. We did not query the participants regarding symptoms or risk factors for infection. However, guidelines were strict in that HCWs were not allowed to work in case of symptoms and it can thus be assumed that the participating HCWs were most likely asymptomatic.

### Study findings in relation to previous research

The present study was performed in parallel with an identically designed study that tested almost 10,000 employees of the Karolinska University Hospital, also in Stockholm, Sweden [10]. The results were highly comparable. In particular, there was also a large variability in the proportion of seropositive subjects among HCWs working in different units of the hospital, with the highest risks for HCWs in emergency wards and acute care wards, and the lowest risks for staff without patient contact [17]. In agreement with our results, many studies have reported an increased risk of exposure to SARS-CoV-2 among HCWs [19], in elderly care homes [12, 15, 20], nursing facilities [16] as well as among the HCWs at hospitals [17, 18, 21]. Seropositivity was comparable to observational studies from hospitals in the same region and near in time [11, 18]. Participating in the active care of COVID-19 patients was a significant risk factor for being seropositive. A similar association between front line COVID-19 care and seropositivity has been observed in a Danish study, screening healthy HCWs, in April 2020 [22]. Occupational exposure to infection has also been found and illustrated as an increased risk of severe COVID-19 among HCWs [19, 23, 24]. In our study, there was a significant decrease of seropositivity with age, which can be due to the that staff with a higher age to a larger extent than otherwise have taken on other tasks than caring for patients with acute infections, during the COVID-19 epidemic. Our findings reveal that especially young HCWs in primary care, care homes and palliative care in Stockholm were highly exposed to SARS-CoV-2. Young individuals have a higher tendency to suffer SARS-CoV-2 infection as asymptomatic COVID-19 disease [25] and thus, young HCWs in primary care may have been a source population of nosocomial infections. The countries that most rapidly achieved a high vaccination coverage i.e. Israel and the UK found vaccination to protect against symptomatic and asymptomatic SARS-CoV-2 infection [26–28]. Asymptomatics and presymptomatics are of particular importance for transmission of SARS-CoV-2 in healthcare settings, as symptomatic HCWs should not be at work. Many countries have prioritized vaccination of HCWs and our data support that maintaining a high degree of immunity among HCWs is crucial to protect vulnerable populations and to safeguard occupational safety and health.

## Conclusion and implications

Our study implies that it would probably have been important with general use of personal protective equipment, systematic testing of suspected cases, contact tracing and isolation of confirmed cases and contacts, and testing of HCWs for SARS-CoV-2 before taking care of vulnerable patients/care takers. The results highlight the exposed situation of healthcare workers in primary care, care homes and palliative care, in a pandemic. This study quantifies the exposure of the healthcare workers during the first wave of the COVID-19 pandemic in the Stockholm Region in Sweden, and sheds light on weaknesses in our crisis and pandemic response readiness. The first wave of the COVID-19 pandemic affected HCWs in the Stockholm region, with cluster outbreaks at workplaces. The occupational health and safety appeared to have been insufficient. Prioritizing sound working environments within healthcare, and prioritizing infection prevention and control including sufficient and adequate personal protective equipment, and vaccination for all HCWs are important to prevent nosocomial infections and to prevent infection as an occupational injury during an ongoing pandemic. Today, a majority of the Swedish HCWs are fully vaccinated against SARS-CoV-2. If emerging variants will be resistant to vaccination the situation could deteriorate and our data imply that maintaining immunity in this group of HCWs is important.

## Supporting information

S1 File IPC measures in PHC reported by operations managers, as examples of how PHC units adapted their work in as a part of the COVID-19 pandemic response. (DOCX)
