# Supplementary material for: Risk for SARS-CoV-2 infection in healthcare workers outside hospitals: A real-life immuno-virological study during the first wave of the COVID-19 epidemic
Source: PLoS One. 2021 Sep 28;16(9):e0257854. doi: 10.1371/journal.pone.0257854 (PMC8478233; doi:10.1371/journal.pone.0257854)
Supplement: S1 File — (DOCX) [file pone.0257854.s001.docx]

**S1 File**

IPC measures in PHC reported by operations managers, as examples of how PHC units adapted their work in as a part of the COVID-19 pandemic response.

Planning and management:

- Rescheduling of physical meetings to digital meetings or telephone calls, or home visits, depending on the patients’ needs.
- Possibility to work from home for staff.
- Triage of patients in the telephone and at the entrances was re-inforced, to make sure patients with symptoms suggestive of COVID-19 were separated from other patients.
- Staff we re-directed to work with new tasks. The need for telephone counselling increased, as did the need for home visits.
- Infection units for patients with suspected COVID-19, and in need of a physical assessment, were started on site or at a nearby site using staff from collaborating PHC units.
- Booked appointments for planned sampling at laboratories (e.g. for medical investigations or planned follow-up visits), in order to minimize crowding in waiting areas.
- Meetings were moved to a larger room to make it easier to keep a safe distance.

On-site infection prevention and control measures (IPC):

- PPE was used as recommended by national guidelines for all patients with symptoms suggestive of COVID-19 and in need of a physical assessment, at infection care units.
- Adjustments of the physical environment with signs indicating appropriate IPC measures for patients such as keeping a safe distance and washing hands.
- Use of PPE: Units started using visirs in *all* patient encounters.
